# Chemical profiling and dermatological and anti-aging properties of *Syzygium jambos* L. (Alston): evidence from molecular docking, molecular dynamics, and *in vitro* experiments

**DOI:** 10.3389/fmolb.2023.1331059

**Published:** 2024-01-05

**Authors:** Ismail Mahdi, Paola Imbimbo, Ahmet Buğra Ortaakarsu, Melvin Adhiambo Ochieng, Widad Ben Bakrim, Badr Eddine Drissi, Mohammed Auwal Ibrahim, Mohamed A. O. Abdelfattah, Mona F. Mahmoud, Daria Maria Monti, Mansour Sobeh

**Affiliations:** ^1^ AgroBioSciences Program, College of Agriculture and Environmental Science, University Mohammed VI Polytechnic, Ben Guerir, Morocco; ^2^ Department of Chemical Sciences, University of Napoli Federico II, Complesso Universitario Monte Sant’Angelo, Napoli, Italy; ^3^ Department of Chemistry, Faculty of Science, Gazi University, Ankara, Türkiye; ^4^ Department of Biochemistry, Ahmadu Bello University, Zaria, Nigeria; ^5^ American University of the Middle East, Egaila, Kuwait; ^6^ Department of Pharmacology and Toxicology, Faculty of Pharmacy, Zagazig University, Zagazig, Egypt

**Keywords:** *Syzygium jambos*, antioxidant, cosmetics, anti-aging, antibacterial, HaCat cells

## Abstract

The phytoconstituents of the aqueous extract from *Syzygium jambos* L. (Alston) leaves were defined using HPLC-PDA-MS/MS and the antioxidant, anti-aging, antibacterial, and anti-biofilm activities of the extract were *in silico* and *in vitro* investigated. The antioxidant activities were performed using *in vitro* DPPH and FRAP assays as well as H_2_-DCFDA assay in HaCaT cells in which oxidative stress was induced by UVA radiation. Anti-aging activity was tested *in vitro*, using aging-related enzymes. The antibacterial, anti-biofilm and inhibitory effects on bacterial mobilities (swarming and swimming) were assessed against *Pseudomonas aeruginosa*. Results showed that *S. jambos* aqueous extract contained 28 phytochemicals belonging to different metabolite classes, mainly phenolic acids, gallic acid derivatives, flavonoids, and ellagitannins. Mineral content analysis showed that *S. jambos* leaves contained moderate amounts of nitrogen, potassium, manganese, magnesium, and zinc, relatively low amounts of phosphorus and copper, and high concentration of calcium and iron. The extract displayed strong antioxidant activities *in vitro* and inhibited UVA-induced oxidative stress in HaCaT cells. Docking the major compounds identified in the extract into the four main protein targets involved in skin aging revealed an appreciable inhibitory potential of these compounds against tyrosinase, elastase, hyaluronidase, and collagenase enzymes. Moreover, molecular dynamic simulations were adopted to confirm the binding affinity of some selected compounds towards the target enzymes. The extract exhibited pronounced *in vitro* anti-aging effects, compared to kojic acid and quercetin (the reference compounds). It also inhibited the growth of *P. aeruginosa*, counteracted its ability to form biofilm, and impeded its swarming and swimming mobilities. Altogether, these findings strongly propose *S. jambos* leaves as a promising source of bioactive metabolites for the development of natural cosmeceutical and dermatological agents.

## 1 Introduction

Given that the skin constitutes the largest organ in the body, it plays a crucial protective role against a variety of chemical, physical, and biological stresses (Jiratchayamaethasakul et al., 2020). Skin aging is a physiological process that is mostly linked to an oxidation-reduction imbalance, caused by the formation of ROS (Reactive oxygen species) and/or a reduction in the ability to scavenge them (Liochev, 2013). Extensive research reported that both external and internal insults generating ROS are related to skin pro-aging effects (Rinnerthaler et al., 2015). The main underlying mechanism involves the disruption of the extracellular matrix (ECM) in the dermis, resulting in the decrease of elastin, collagen, melanin and hyaluronic acid responsible for skin health and rejuvenation (Rinnerthaler et al., 2015).

In the human body, free radicals are derived from reactive oxygen (ROS) and nitrogen (RNS) species and are generated by several endogenous systems, pathophysiological states and exposure to different stressful conditions (Michalak and Kasztelan-Szczerbińska, 2022). The imbalanced production (excessive or inefficient detoxification) of ROS inflicts oxidative damage which has an impact on cellular structures (Comino-Sanz et al., 2021). These free radicals were reported as key factors implicated in aging, wound healing, and other human pathological conditions (Fitzmaurice, Sivamani and 2011; Michalak and Kasztelan-Szczerbińska, 2022).

The growing interest in natural molecules to counteract skin aging is rooted in the quest for safer and sustainable alternatives to conventional and synthetic molecules. Conventional anti-aging strategies often show side effects, prompting a shift in consumer preferences towards natural products. This evolving scenario reflects a broader societal movement towards holistic wellbeing and environmentally friendly practices. In this context, exploring the efficacy of natural compounds in skincare solutions aligns with the current consumer trends and provides insights that are relevant for the development of safe, effective, and eco-friendly skincare solutions (Ahmed et al., 2020; Mahdi et al., 2022). Natural antioxidants have been found to strengthen the endogenous antioxidant defenses and help the optimal balance restoration. In addition, numerous studies have demonstrated the antioxidant properties of phenolics linked to different mechanisms, including free radical-scavenging, hydrogenation, quenching singlet oxygen, chelation of metal ions, and reacting with several reactive species such as hydroxyl and superoxide (Adwas et al., 2019).

*Syzygium jambos* L. (Alston), commonly recognized as rose apple, is a medium-sized medicinal plant belonging to the family Myrtaceae, and is widespread in sub-Saharan Africa, Central America, and Asia. Moreover, several studies have highlighted the pharmacological activities of its various parts such as antibacterial, antioxidant, anticancer, anti-inflammatory, antidiabetic, and hepatoprotective (Bonfanti et al., 2013; Chua et al., 2019; Ochieng et al., 2022). *Syzygium jambos* leaf extracts have been found to encompass various bioactive phytochemicals such as phenolic acids, flavonoids, phloroglucinols, and ellagitannins (Ávila-Peña et al., 2007; Chua et al., 2019; Ochieng et al., 2022). Some *Syzygium* species have been reported for their potential application as cosmetic ingredients (Palanisamy et al., 2011; Sharma, 2014). However, there are no reports, so far, on the anti-aging potential and skin health-promoting effect of S. jambos. Thus, this study aims to profile the phytochemical composition of the aqueous leaf extract of S. jambos and investigate its cytotoxic, antioxidant and anti-aging properties using *in vitro* and cell-based approaches. Additionally, we assessed the *in vitro* antibacterial, anti-biofilm, anti-swimming, and anti-swarming effects of the plant extract against *Pseudomonas aeruginosa*, which is a relevant bacterial strain in skincare treatment due to its association with antibiotic-resistant skin infections, emphasizing the importance of understanding the inhibitory effects of the extract against this bacterium (Spernovasilis et al., 2021). In addition, as minerals in medicinal plants often work in synergy with other bioactive compounds, the mineral content of S. jambos leaves was also determined to expand our understanding of its nutritional characteristics and its potential as a valuable source of minerals that are crucial for supporting skin health and preventing premature aging (Jung et al., 2009; Mukherjee et al., 2011). We further employed molecular docking to assess the interaction of key compounds identified in the extract with the four primary enzyme targets associated with the skin aging process. Moreover, molecular dynamic simulations were adopted to monitor the binding affinity of some selected compounds towards the target enzymes.

This study showed that the aqueous extract of *S. jambos* contained a diverse range of phytochemicals, including phenolic acids and flavonoids, along with some essential mineral elements. It exhibited strong antioxidant properties, countered UVA-induced oxidative stress, demonstrated notable inhibitory effects on skin aging-related enzymes, and inhibited *P. aeruginosa* growth, biofilm formation, and motility. These findings demonstrate the efficacy of *S. jambos* in countering UVA-induced oxidative skin damage, preventing skin aging, and inhibiting skin infections. Nevertheless, further experiments involving fractionation, guided bioassays and animal models would be paramount to confirm these findings and provide scientifically sound information on the potential application of *S. jambos* as a valuable natural resource with diverse benefits for skin health and anti-aging interventions.

## 2 Materials and methods

### 2.1 Plant material and extraction, phytocontents and LC-MS/MS analysis

*Syzygium jambos* L. (Alston) leaves were gathered from trees grown in private garden, Zaria, Nigeria in 2021, air-dried, powdered, and subjected to ultrasound-assisted extraction in distilled water (15 g x 200 mL). The extraction process was performed at 20 kHz, 5°C, and an amplitude of 30%. The resulting extract was filtered, dehydrated using a rotary evaporator and the extraction yield was calculated (mass of extract/mass of dry matter × 100) (Extraction yield of 27.2%). To determine the total phenolic content (TPC) of the extract, the Folin-Ciocalteu (FC) technique was utilized, and measurements were conducted in a 96-well microplate (Dicko et al., 2002). The total flavonoid content (TFC) was measured using the aluminum chloride colorimetric method, with slight adjustments made to adapt it for a 96-well plate (Chang et al., 2002). To analyze the phytochemical composition of *S. jambos* leaf extract, we used an HPLC-PDA-MS/MS system, which included a Shimadzu Japan system (Tokyo, Japan) connected to an MS 8050 mass spectrometer featuring an electrospray ionization (ESI) source (Mahdi et al., 2023). The separation process was conducted on a C18 reversed-phase column (Zorbax Eclipse XDB-C18, 4.6 × 150 mm, 3.5 μm, Agilent). A gradient of water and acetonitrile (ACN) with 0.1% formic acid each ranged from 5% to 30% ACN over 1 h, at a flow rate of 1 mL/min. Automated sample injection was carried out with an autosampler SIL-40C xs. The instrument was operated using LC solution software (Shimadzu), and MS operated in the negative full scan mode.

### 2.2 Mineral content analysis

The mineral composition of *S. jambos* leaves was determined by multi-elemental trace analysis using the Agilent 5110 ICP-OES (Inductively Coupled Plasma Optical Emission Spectroscopy) (Santa Clara, California, United States). The analyzed elements were potassium (K), phosphorus (P), magnesium (Mg), calcium (Ca), sodium (Na), iron (Fe), zinc (Zn), manganese (Mn), and copper (Cu) (Mahdi et al., 2023). In addition, the total nitrogen content was analyzed according to the Kjeldahl method (Baker et al., 1992).

### 2.3 *In vitro* antioxidant and anti-aging evaluation

The antioxidant activity was estimated using DPPH (BLOISMARSDEN, 1958) and FRAP assays (Du and Xu, 2014), established as previously reported (Ghareeb et al., 2018). The skin anti-aging activities were evaluated *in vitro* using four skin enzymes involved in the process of skin aging, namely, collagenase, tyrosinase, elastase and hyaluronidase. These assays were carried out as previously described in (Bakrim et al., 2022).

### 2.4 Cell culture and MTT assay

Human epidermal keratinocytes (HaCaT) (Innoprot, Biscay, Spain) were used to assess the potential cytotoxicity of the extract. The cells were maintained in Dulbecco’s Modified Eagle’s Medium supplemented with 10% foetal bovine serum (FBS) and 2 mM L-glutamine and antibiotics, in a 5% CO_2_ humidified atmosphere at 37°C (Petruk et al., 2016). Cells sub-culture was carried out every 72 h in a ratio of 1:4. For this purpose, after removing the culture medium, cells were rinsed with PBS and detached with trypsin/EDTA. Cells were then collected in fresh complete growth medium, centrifuged and sub-cultured. For cell viability assay, cells were plated into a 96-well microplate at a density of 2 × 10^3^/well. After 24 h, the extract was added at increasing concentrations (25–100 μg/mL) and incubated for 48 h. The percentage of viable cells (expressed as the percentage of viable cells in the presence of the extract compared to controls) was determined using the MTT assay (Monti et al., 2015). Two groups of cells were used as control, i.e., untreated cells and cells supplemented with identical volumes of water. The average of the two control groups was used as 100%.

### 2.5 Oxidative stress assessment using UVA radiation

To examine the extract’s ability to counteract intracellular oxidative stress, HaCaT cells (2 × 10^4^ cells/cm^2^) were seeded and 50 μg/mL of the extract was added to the cells 24 h later and incubated for 2 h. After incubation with the extract, cells were washed twice with cold phosphate-buffered saline (PBS), incubated with PBS, and then irradiated by Ultraviolet A (UVA) rays. Cells were exposed to UVA radiation by using a commercial lamp (UVItec, Cambridge, United Kingdom; 4 × 9W lamps, λmax, 365 nm, no detectable emission below 320 nm) at a dose of 100 J/cm^2^ (which corresponds to 10 min of irradiation by the used apparatus). Immediately after stress induction, ROS production and GSH (Glutathione) levels were estimated using DCFDA (Petruk et al., 2016) and DTNB assays (Liberti et al., 2023), respectively.

### 2.6 Antibacterial activities

#### 2.6.1 MIC determination

The MIC (Minimum Inhibitory Concentration) of *S. jambos* aqueous extract was assessed against *P. aeruginosa* by the microdilution method (Abbas et al., 2017; Bakrim et al., 2022). The extract was dissolved in Muller–Hinton (MH) broth to obtain a final concentration of 100 mg/mL. Next, the extract was sterilized using 0.22 *μ*m syringe filters and two-fold serially diluted into a sterile microplate’s wells in triplicate (1.562–100 mg/mL). Subsequently, a fresh bacterial suspension of *P. aeruginosa* (OD_600nm_ = 0.6) was introduced into each well (2 *μ*L/well). Uninoculated wells were established as negative controls. In addition, media without extract amendment were used as growth controls and uninoculated MH media were established as blank controls. The microplate was incubated at 37°C, in shaker at 150 rpm and the bacterial growth was visually and spectrophotometrically assessed after 18 h incubation. The MIC was determined as the minimum concentration of the extract that prevented the observable bacterial growth. The Tukey’s *post hoc* test was performed to analyze the significant differences between treatments.

#### 2.6.2 Biofilm inhibition assay

The effects of the sub-MIC concentrations [1/8 MIC (12.5 mg/mL) and 1/4 MIC (25 mg/mL)] of *S. jambos* extract on the biofilm-forming ability of *P. aeruginosa* were studied using the crystal violet colorimetric assay in a 96-well microplate (Mostafa et al., 2020; Mahdi et al., 2021). The inoculation was performed as described above in the MIC assay. At the end of the incubation, the bacterial suspensions were discarded, and the wells were washed twice with a phosphate-buffered saline (PBS) and adherent bacteria were stained by a 1% crystal violet (CV) solution. Next, CV solutions were discarded, and wells were washed with sterile distilled water to eliminate the excess dye. The attached biofilm was solubilized by filling each well with 95% ethanol. The amount of biofilm in each media was estimated spectrophotometrically at OD_595 nm_. The Tukey’s *post hoc* test was performed to analyze the significant differences between treatments.

#### 2.6.3 Swimming and swarming assessment on plates

*Syzygium jambos* aqueous extract at the sub-MIC concentrations was used to assess the extract’s effect on the swimming and swarming motilities of *P. aeruginosa* (Mostafa et al., 2020; Tawfeek et al., 2023). The swimming and swarming media were prepared (Yeung et al., 2009), cooled (<50°C), and supplemented with the filtered extracts to obtain final doses of 1/8 and 1/4 MICs. Next, 10 μL of a fresh suspension of *P. aeruginosa* (OD_600 nm_ = 0.6) was deposited in the middle of the agar plates and incubated at 37°C for 24 h. The mobilities zone diameters were measured in cm. The Tukey’s *post hoc* test was performed to analyze the significant differences between treatments.

### 2.7 Molecular docking

The molecular docking was performed using the molecular operating environment software (MOE; MOE2022. v11.18.1) as per the protocol detailed in (Bogari et al., 2022). In brief, the enzymes’ structures were downloaded from protein data bank (www.pdb.org) with the corresponding bound ligand inhibitors. The protein data bank (PDB) codes were: elastase (PDB id: 1Y93), Tyrosinase (PDB id: 2Y9X), hyaluronidase (PDB id: 1FCV), and collagenase (PDB id: 2D1N). The Quickprep panel of the software was used to prepare the proteins’ structures by deleting the unbound water molecules, protonation, and energy minimization. All ligands were downloaded from PubChem and saved as (.sdf) files, energy minimized, and compiled into a database together with the co-crystalized ligand inhibitors. Then, the database wash tool of the software was used to adjust the ligands’ formal charges for strong acids and bases and to adjust bond lengths’ scales. The docking protocol used the default parameters as placement method, triangle matcher, and London dG scoring function. For validation, each co-crystallized ligand inhibitor was docked to the corresponding protein and the RMSD values were all less than 2Å.

### 2.8 Molecular dynamics

Maestro Software Package (2023-2) of Schrodinger Software (Schrödinger, Schrödinger Release 2023-2: Maestro, Schrödinger, LLC, Maestro-Desmond Interoperability Tools, Desmond Molecular Dynamics System) was used for all operations related to molecular dynamics (MD) study.

#### 2.8.1 Protein preparation

The Protein Preparation Wizard tool in Maestro was used for protein preparation. These processes were performed separately for tyrosinase, hyaluronidase, elastase, and collagenase enzyme protein structures to be simulated MD. Protein preparation was carried out at physiological pH 7.4 with and the process included the calculation of the ionization states of the protein structures. Hydrogen bonds in the protein structure were optimized according to pH 7.4. The protein preparation process ended with the minimization of protein structures using the OPLS3e force field (Harder et al., 2016; Roos et al., 2019).

#### 2.8.2 System setup

The System Builder tool in Maestro was used for the system setup. Protein structures were immersed in an orthorhombic solvent box. The solvent boxes were prepared to contain the entire protein structure and were prepared in 15 Å × 15 Å x 15 Å dimensions. The solvent box consisted of water molecules using the TIP3P (Mark and Nilsson, 2001) water model and 0.15 M NaCl was added to the solvent boxes to ensure accurate modelling of the physiological environment at specific constant temperature. Additional sodium ions were added as needed to keep the system neutral. System setup was performed using OPLS3e force field.

#### 2.8.3 MD simulations

All MD simulations were performed with the Desmond module in the Maestro Software Package (Maestro-Release, 2023). The NPT option was employed as it was desired to include constant temperature, constant pressure and constant particle number for each of the MD simulations. Nose-Hoover thermostat was used to keep the temperature of the system constant (Evans and Holian, 1985). The temperature of the system was kept at 300 K throughout the simulations. The pressure of the system was kept at 1 bar throughout the simulations using Martyna-Tobias-Klein barostat (Martyna et al., 1994). All these adjustments enabled high-precision temperature and pressure control of the environment in the MD simulation and produced data with more realistic results. All the simulations were created using the 2 ps relaxation protocol. Following these adjustments, the duration of the MD simulation was set to 200 ns for both apo (ligand free) and halo forms (ligand containing) and 2000 frames were requested for each simulation (Ucuncu et al., 2023).

#### 2.8.4 Generalized born surface area (MM-GBSA) calculation

In order to determine the stability of the complexes obtained and to investigate the state of their free energies, the Generalized Born Surface Area (MM-GBSA) was calculated and graphed every 400 frames in 200 ns MD simulations (Rastelli et al., 2010; Nada et al., 2022). MM-GBSA calculations provide numerical prediction of the interaction mechanisms of complexes, and obtaining relatively low values is interpreted as a stable complex. Monitoring the change of free energy data, which gives an idea about the stability of the complex, throughout molecular dynamics’ simulation provides important inferences about whether it is possible to maintain the stability of the created complexes. For this purpose, MM-GBSA in Maestro was used to determine the complex binding free energy. MM-GBSA calculations were performed using the VSGB (Li et al., 2011) solver model. OPLS3e force field was employed to obtain accurate results and to be compatible with the method used in MD system setup. This calculation was made using the Prime module, including the rotamer search algorithm, and used the following formula (Xu et al., 2013):

ΔG = Ecomplex (minimized)–[Eligand (minimized) + Ereceptor (minimized)].

### 2.9 Statistical analysis

Each experiment was done in triplicates and the results were expressed as mean ± SD. The statistical analysis was performed using one-way analysis of variance (ANOVA) followed by Bonferroni test (*post hoc*). The statistically significant differences were set at *p* < 0.05.

## 3 Results and discussion

### 3.1 Phytochemical composition of the aqueous extract of *S. jambos* leaves by HPLC-PDA-MS/MS

**FIGURE 1:**
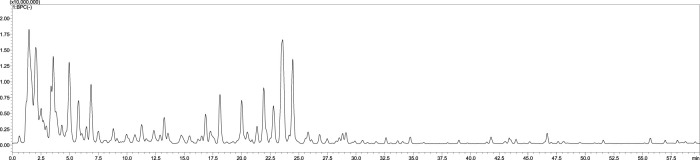
The LC-MS chromatogram of *S. jambos* leaves aqueous extract.

The LC-MS chromatogram of the aqueous extract of *S. jambos* leaves is shown in Figure 1. The compounds annotated via LC-MS/MS analysis in the extract are listed in Table 1. The analysis revealed the presence of 28 phytochemicals belonging to different metabolite families including phenolic and organic acids, flavonoids, and ellagitannins. The compounds were identified based on their retention times (RT), UV spectra, molecular weights, and confirmed using the available authentic compounds. These findings agree with those reported from the Egyptian flora (Sobeh et al., 2018a). Also, other *Syzygium* species showed similar chemical patterns, among them *Syzygium cumini, Syzygium samarangense, Syzygium aqueum*; they are polyphenols-rich medicinal plants containing myricetin and gallic acid (Sobeh et al., 2018b; Chagas et al., 2018; Sobeh et al., 2018c; Gaspar et al., 2020; Yassir et al., 2022).

**TABLE 1:** Annotated phytoconstituents of the aqueous extract from *S. jambos* leaves using HPLC-MS/MS analysis.

Retention time (min)	[M-H]-	MS/MS	Identified compounds
1.63	133	115	Malic acid
1.99	191	111	Citric acid
2.00	481	301	2,3-(S)-Hexahydroxydiphenoyl-*D*-glucose
2.47	331	169	Galloyl glucose
3.31	169	125	Gallic acid
3.32	343	191	Galloylquinic acid
3.74	435	169	Benzoyl galloyl glucose
4.46	449	169	Phenylacetyl galloyl glucose
5.72	305	125	(epi)Gallocatechin
6.26	153	108	Dihydroxybenzoic acid
8.96	295	163	Coumaric acid pentoside
10.83	373	169	Acetyl-*O*-galloyl glucose
11.13	353	191	Chlorogenic acid^$^
12.83	483	169	Digalloyl glucose
13.83	469	301	Valoneic acid dilactone
15.04	631	317	Myricetin glucoside gallate
17.99	479	317	Myricetin glucoside
19.42	493	317	Myricetin glucuronide
20.27	449	317	Myricetin pentoside
21.29	477	301	Quercetin glucuronide
22.92	433	301	Quercetin pentoside
23.27	595	317	Myricetin xylosyl-rhamnoside
24.35	433	301	Quercetin pentoside
26.76	447	301	Quercetin rhamnoside
26.94	447	301	Quercetin rhamnoside
28.20	461	299	Diosmetin glucoside
28.80	417	285	Kaempferol pentoside

### 3.2 Mineral content

Minerals in medicinal plants play a crucial role in their therapeutic effectiveness and overall health benefits. For instance, calcium, magnesium, zinc, potassium, and iron are vital for maintaining proper body functions, including bone health, muscle contraction, nerve transmission, and immune system function (Mahdi et al., 2023). In addition, minerals are also crucial in maintaining optimal skin health and function as they contribute to the overall structure, appearance, and function of the skin (Mahdi et al., 2023). Calcium, for instance, promotes cell renewal and contributes to the skin’s barrier function, ensuring adequate moisture retention and protection against external aggressors. Zinc is another important mineral that aids in wound healing, controls sebum production, and helps combat acne and other skin conditions. Magnesium supports cellular energy production, thereby contributing to a healthy and vibrant complexion. Copper aids in collagen synthesis, enhancing skin elasticity and firmness. Iron is vital for oxygen transportation to skin cells (Kollaras, 2014; Park, 2015; Vollmer, West, 2018). Therefore, by incorporating a balanced diet rich in minerals or using skincare products containing mineral-rich ingredients, individuals can help promote radiant, nourished, and resilient skin. To exert their beneficial effects, minerals in plants often work in synergy with other bioactive compounds, such as vitamins and phytochemicals. Hence, harnessing the mineral content in medicinal plants is essential for maximizing their therapeutic properties and promoting holistic health and wellbeing. In this study, we showed that, according to the typical ranges of mineral contents in plants and previous studies addressing the nutraceutical merit of *S. jambos* L. (Rajalakshmi et al., 2016), the plant’s leaves contained moderate levels of nitrogen, potassium, magnesium, manganese, and zinc, relatively low contents of phosphorus and copper, and high calcium and iron concentrations (Table 2). Comparatively, Rajalakshmi et al. (2016) reported that the leaf extract of *S. jambos* contains adequate amounts of sodium (1.62% DW), potassium (4.29% DW), iron (2.38% DW), calcium (8.33% DW), magnesium (0.96% DW) and phosphorus (3.31% DW). Another study revealed that both the fruits and seeds of *S. jambos* contained sufficient quantities of important macro and micro minerals including calcium (12.71 and 23.72 mg/100 g, respectively), iron (0.73 and 1.15 mg/100 g, respectively), phosphorus (11.63 and 14.62 mg/100 g, respectively), zinc (0.24 and 0.39 mg/100 g, respectively), potassium (45.62 and 329.32 mg/100 g, respectively), manganese (0.045 and 0.093 mg/100g, respectively) and copper (0.067 and 0.186 mg/100 g, respectively) (Dutta and Khaled, 2021). Noteworthy, the plant tissues, especially the seeds, are remarkably rich in calcium, phosphorus, potassium, and copper. Altogether, these investigations show that *S. jambos* has the potential to serve as a valuable source of essential minerals to fulfill daily dietary needs and support skin-health-promoting effects.

**TABLE 2:** Mineral composition of *S. jambos* leaves.

Parameters	Unit	Concentration	Method
Total nitrogen (N)	% of DW	1.13	Kjeldahl
Total phosphorus (P)		0.14	ICP-EOS
Potassium (K)		1.06	
Calcium (Ca)		1.23	
Magnesium (Mg)		0.35	
Sodium (Na)	mg/kg DW	287.35	
Iron (Fe)		517.10	
Manganese (Mn)		26.95	
Zinc (Zn)		18.34	
Copper (Cu)		7.19	

DW: dry weight, ICP-EOS: inductively coupled plasma optical emission spectroscopy.

### 3.3 Molecular docking

An *in silico* molecular docking approach was adopted to get clear insights about the binding modes of the major extract’s constituents towards collagenase, elastase, tyrosinase, and hyaluronidase enzymes, and to estimate their binding affinity potential as well. Herein, thirteen major compounds identified in *S. jambos* aqueous extract, belonging to phenolic acids, flavonoids, and glucose derivatives, were docked to the aforementioned target enzymes (Table 3). Noteworthy, we previously characterized the other extract’s components in *Euphorbia retusa* leaf extract and docked them to these target enzymes (Elgamal et al., 2021). Docking results revealed conspicuous binding affinity of the docked compounds towards the four enzymes, which was indicated by the docking score values and the various interactions afforded between the extract’s compounds and the amino acid residues framing the binding sites of the target enzymes.

**TABLE 3:** Docking scores of the major compounds identified in *S. jambos* aqueous extract upon docking into skin aging target enzymes.

Compound name	Docking score (kcal/mol)			
	Elastase	Tyrosinase	Hyaluronidase	Collagenase
	(1Y93)	(2Y9X)	(1FCV)	(2D1N)
Dihydroxybenzoic acid	−18.24	−18.15	−10.65	−13.61
Theogallin	−24.35	−18.84	−16.17	−18.59
Coumaric acid pentoside	−12.35	−18.74	−14.88	−15.47
Kaempferol pentoside	−18.43	−11.53	−17.26	−14.83
Diosmetin glucoside	−14.10	−12.49	−13.39	−16.27
Quercetin rhamnoside	−18.39	−13.96	−17.41	−16.62
Myricetin pentoside	−19.49	−13.91	−20.27	−19.82
Myricetin glucuronide	−23.26	−20.30	−22.75	−23.61
Myricetin xylosyl rhamnoside	−23.09	−13.09	−20.58	−18.71
Digalloyl glucose	−23.69	−11.79	−21.15	−17.46
Acetyl-*O*-galloyl glucose	−16.26	−12.87	−16.19	−13.17
Phenyl acetyl galloyl glucose	−19.24	−11.25	−16.34	−14.75
Benzoyl galloyl glucose	−22.20	−11.55	−16.73	−14.65
Quercetin	–	–	–	−16.32
Kojic acid	−9.49	−8.15	−12.66	–

**FIGURE 2:**
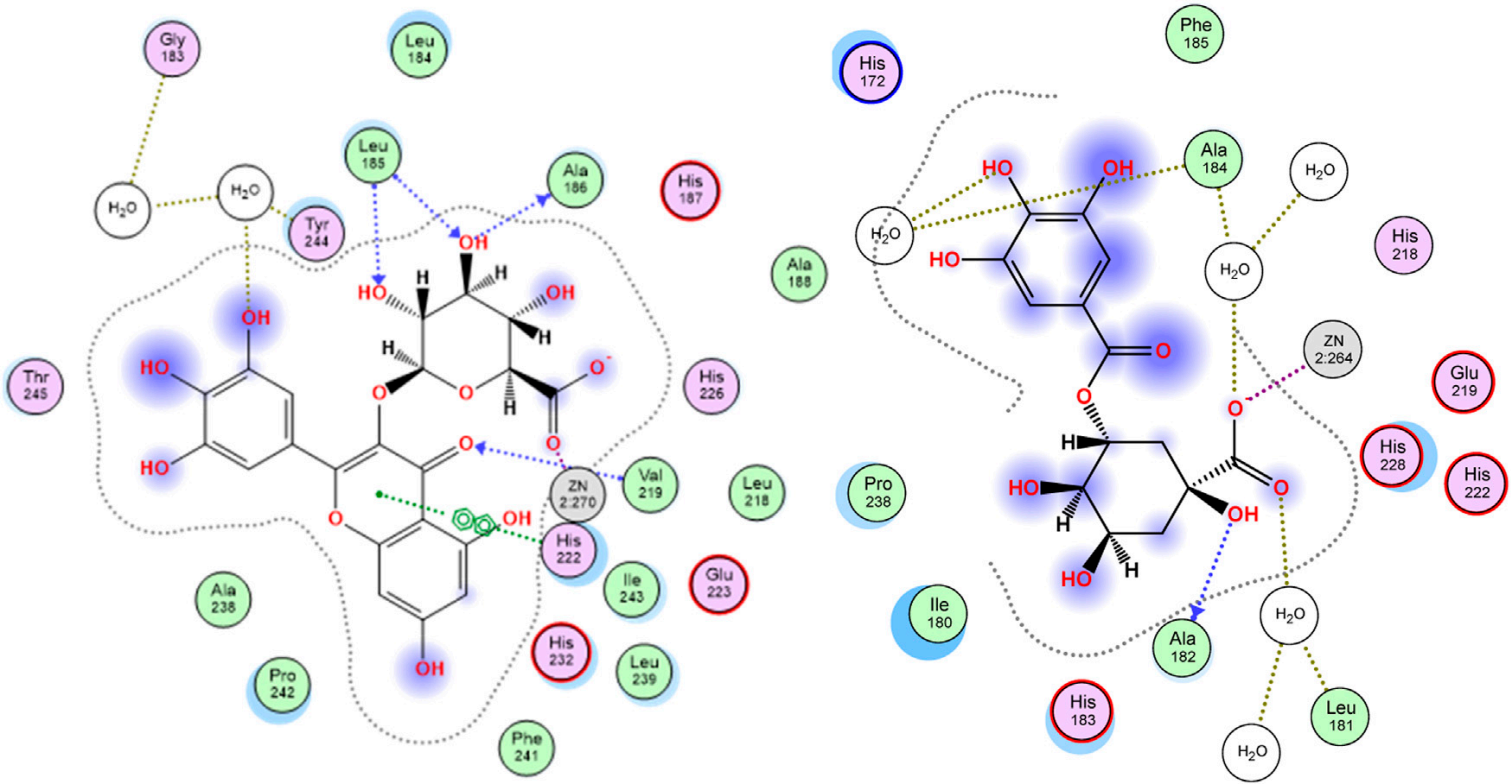
2D docking poses of myricetin glucuronide (left) and theogallin (right) docked in the active site of collagenase and elastase enzymes, respectively.

The enzyme collagenase, or MMP3, belongs to the super enzyme family of metalloproteinases and breaks the peptide linkages in collagen leading to its degradation. Being a metalloprotease, the binding site of the enzyme has Zn^2+^ ion and is outlined by the amino acid residues Leu185, Ala186, Ala188, Val219, His222, Glu223, Pro242, Tyr244, and Thr245 (Kohno et al., 2006). Docking *S. jambos* aqueous extract’s major compounds to collagenase showed that the compounds fitted properly in the enzyme’s active site and afforded comparable docking scores relative to the reference inhibitor, quercetin, which estimates appreciable binding affinities of the docked compounds. Noteworthy, six compounds showed lower docking scores (better binding affinity) than quercetin (Table 3). Myricetin glucuronide showed the best docking score (−23.61 kcal/mol) among the flavonoids and all other compounds, whereas theogallin (−18.59 kcal/mol) and digalloyl glucose (−17.46 kcal/mol) were the superior compounds in the phenolic acids class and the glucose derivatives, respectively. Figure 2 depicts the interactions afforded between myricetin glucuronide and the amino acid residues in collagenase active site, which included Zn^2+^ ion chelation through the carboxylate moiety of the ligand, hydrogen bonds with Leu185, Ala186, and Val219, and pi-pi interaction with His222.

Similarly, elastase, MMP12, is another member of the metalloproteinase enzymes family that degrades the elastin protein in the extracellular matrix (ECM) of the skin via cleaving the peptide linkages. The active site of the enzyme is marked by the Zn^2+^ ion and outlined by the residues His172, Gly179, Ala182, His183, Ala184, Glu219, His222, His228, and Pro238. The chelation of the zinc ion and the interactions with Ala184 and Glu219 are among the reported interactions of elastase inhibitors (Bertini et al., 2005). Interestingly, all the docked compounds of *S. jambos* extract showed to surpass the reference elastase inhibitor, kojic acid, with respect to the docking scores, where the extract’s compounds had docking score values in the range of −12.35 to −24.35 kcal/mol *versus* −9.49 kcal/mol for kojic acid (Table 3). Theogallin showed the best docking score (−24.35 kcal/mol) among the phenolic acids and all other compounds, whereas myricetin glucuronide (−23.26 kcal/mol) and digalloyl glucose (−23.69 kcal/mol) were the superior compounds in the flavonoids class and the glucose derivatives, respectively. Theogallin was able to chelate the Zn^2+^ ion in the active site of elastase through its carboxylate moiety and afforded hydrogen bonds with Leu181, Ala182, and Ala184, Figure 2.

**FIGURE 3:**
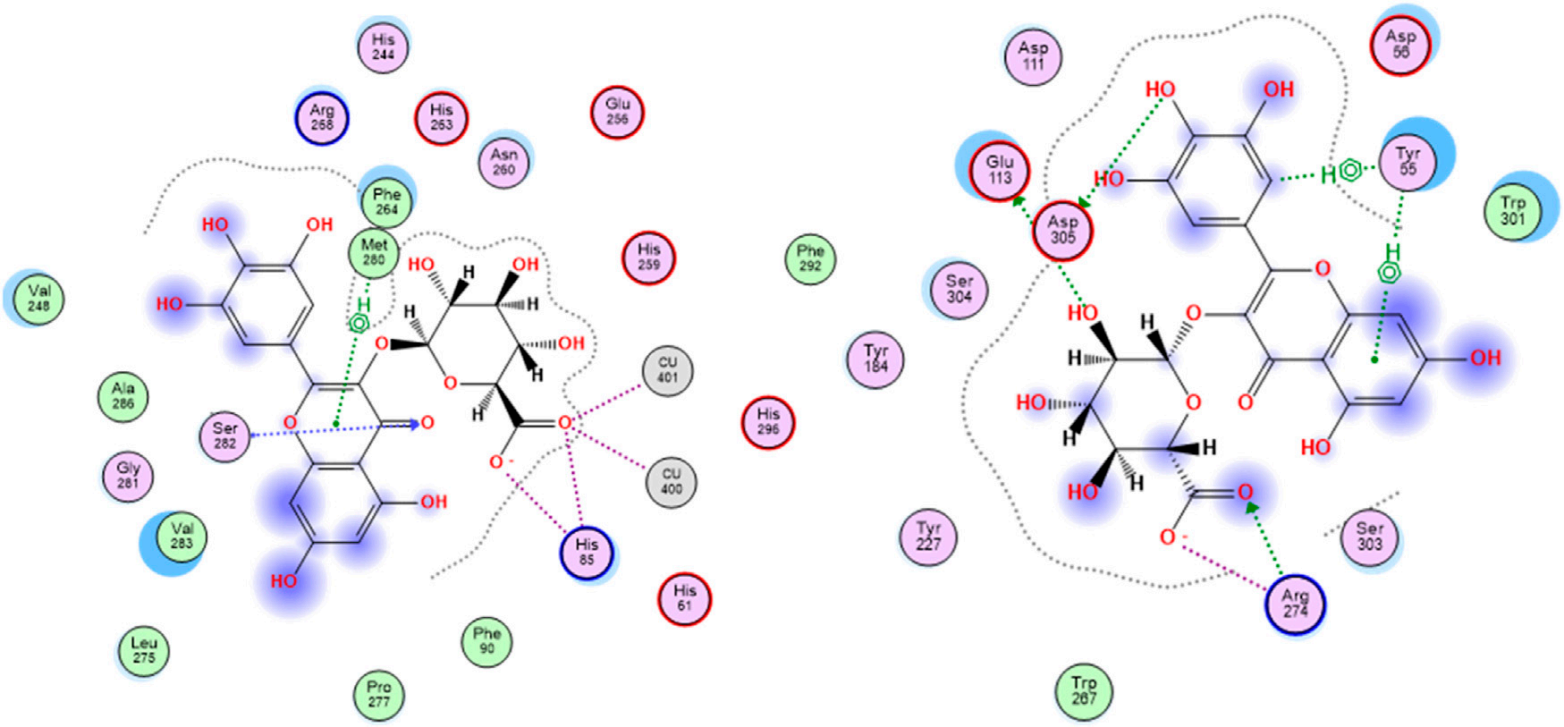
2D docking poses of myricetin glucuronide docked in the active site of tyrosinase (left) and hyaluronidase (right) enzymes.

Tyrosinase is a metallo-enzyme involved in the production of the pigment melanin in the melanocytes by catalyzing the hydroxylation of tyrosine to 3, 4-dihydroxyphenylalanine (L-DOPA) that is in turn oxidized to the melanin precursor, dopaquinone. Hyperpigmentation is one of the conspicuous signs of skin aging, thus blocking tyrosinase activity is among the effective mechanisms of anti-aging agents. The active site of the enzyme is characterized by the presence of two tetragonal Cu^2+^ ions and outlined by the amino acid residues Asn81, His85, Asn260, His263, Phe264, Arg268, Met280, Val283, and Glu322. Concerning ligand-tyrosinase interactions, effective tyrosinase inhibitors are reported to afford copper ions chelation and interact with Asn260, His263, and Met280 residues in the enzyme’s active site (Ismaya et al., 2011). All the *S. jambos* extract compounds had better docking scores (−20.30 to −11.25 kcal/mol) than the reference inhibitor, kojic acid (−8.15 kcal/mol), when docked to tyrosinase enzyme, indicating their high inhibitory potential against the enzyme (Table 3). The highest binding affinity towards tyrosinase among the flavonoids and all other compounds was shown by myricetin glucuronide, which had the lowest docking score value (−20.30 kcal/mol), whereas theogallin (−18.84 kcal/mol) and acetyl-*O*-galloyl glucose (−12.87 kcal/mol) were the top compounds in the phenolic acids class and the glucose derivatives, respectively. At the enzyme’s active site, myricetin glucuronide was able to chelate both Cu^2+^ ions by virtue of its carboxylate moiety. Moreover, it afforded hydrogen bond interactions with His85 and Ser282, ionic interaction with His85, and sigma-pi interaction with Met280, Figure 3.

The glycosaminoglycan derivative, hyaluronic acid, is among the crucial components of the ECM in the skin. It maintains the skin’s elasticity and moisture, mediates tissues’ remodeling and repair, and helps with the transportation of both nutrients and waste materials. Degradation of hyaluronic acid is catalyzed by the enzyme hyaluronidase, which is naturally synthesized during the aging process. The enzyme cleaves the linkages between the N-acetylglucosamine and glucoronate units. The ligand-hyaluronidase enzyme crystal structure reveals polar and non-polar interactions with the amino acid residues Tyr 55, Glu 113, Tyr 184, among others (Marković-Housley et al., 2000). Out of the docked *S. jambos* extract’s compounds, nine were able to surpass the reference inhibitor, kojic acid, with respect to the binding affinity to the enzyme as they had docking score values between −16.19 and −22.75 kcal/mol *versus* −12.66 kcal/mol for kojic acid. However, diosmetin glucoside and coumaric acid pentoside were slightly better than kojic acid with scores of −13.39 and −14.88 kcal/mol, respectively, while dihydroxybenzoic acid had the docking score, −10.65 kcal/mol, which indicates lower binding affinity than the reference drug (Table 3). The highest binding affinity towards hyaluronidase enzyme among the flavonoids and all other compounds was shown by myricetin glucuronide, which had the lowest docking score value (−22.75 kcal/mol), whereas theogallin (−16.17 kcal/mol) and digalloyl glucose (−21.05 kcal/mol) were the top compounds in the phenolic acids class and the glucose derivatives, respectively. Myricetin glucuronide afforded several interactions at the enzyme’s active site including hydrogen bond interactions with Glu113, Arg274, Asp305, ionic interaction with Arg274, and two sigma-pi interactions with Tyr55, Figure 3. The presented results highlighted a significant inhibitory potential of myricetin glucuronide, theogallin, and digalloyl glucose, identified in *S. jambos* leaves aqueous extract, against four target enzymes with significant roles in skin aging. Each of these compounds showed the best binding affinity, among its class members, towards all the employed enzymes.

### 3.4 Molecular dynamics

#### 3.4.1 Root-mean-square deviation (RMSD) analysis

**FIGURE 4:**
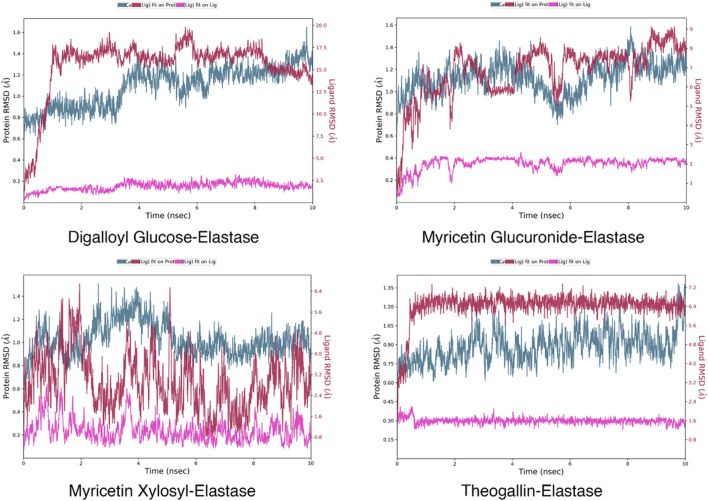
RMSD plots of the ligand-elastase complexes in 200 ns MD simulations.

In molecular docking studies, the fact that the docking scores of the ligands docked to elastase enzyme were remarkably close to each other required preliminary MD simulations to find the main compound that stands out in the inhibition of elastase enzyme in the plant extract (Alonso et al., 2006). For this reason, 10 ns molecular dynamics simulations of the complexes formed by the elastase and the compounds digalloyl glucose, myricetin glucuronide, myricetin xylosyl and theogallin were successfully conducted. The root-mean-square deviation (RMSD) graphs indicate how much the average position changes relative to a particular frame during the MD simulation. The graphs were calculated based on the positions of alpha carbons and compared with each other. Compound structures that show the least RMSD change and form a plateau tend to form more stable complexes with the corresponding protein. Accordingly, theogallin-elastase complex was identified as the lead compound with low RMSD values and extremely stable plateau level and was used in the 200 ns long MD simulation, Figure 4.

**FIGURE 5:**
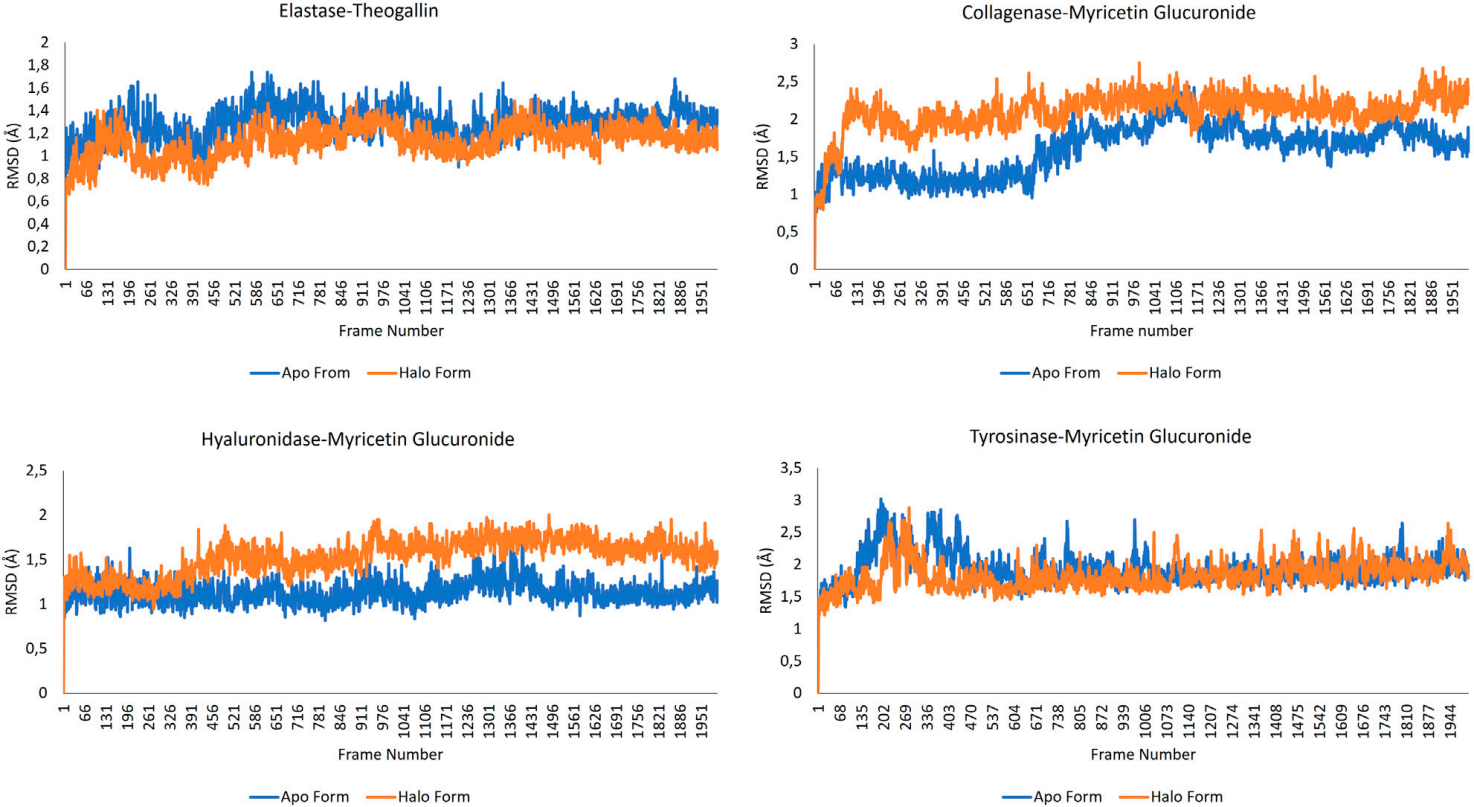
RMSD data for the complexes formed by the lead compounds and the different protein structures.

RMSD data are particularly useful for understanding conformational constraints and distortions in protein structure. In cases where the halo form plot is below that of the apo form, the ligand is meant to cause a conformational constraint. On the contrary, when the halo form plot is above that of the apo form, it indicates that the ligand causes a conformational differentiation in the protein structure such that the protein structure cannot fulfil its function. RMSD data resulting from MD simulation of the complexes formed by the lead compounds and the same protein structures without any ligands are shown in Figure 5.

Viewing the RMSD data of the elastase-theogallin complex, and the movement of the elastase protein structure alone, it was noticed that RMSD graphs differ from each other. This difference between halo form and apo form indicates that theogallin compound causes a conformational restriction on elastase. The graphs moved in the same direction from the beginning to the end of the simulation, but the halo form graph had lower RMSD values. This is an indication that the halo form tends to make the same movement, but its movement is limited. This limitation in the natural movement of the protein structure is a noticeably clear sign of strong inhibition. The apo form and halo form graphs form a plateau level. This plateau level consisting of repeating structures indicates that theogallin compound will continue to inhibit the enzyme.

Examining the RMSD data of myricetin glucuronide-collagenase complex, and that of the natural movement of the collagenase protein structure, it was noticed that the values are quite different from each other. The halo form graph remained well above the apo form graph at all times of the simulation and created a noticeable difference. This difference shows that the natural movement of the protein structure is disrupted. This means that the average position of the protein collagenase underwent exceptionally large changes indicating very strong inhibition of the enzyme activity.

As for the hyaluronidase enzyme, the RMSD graphs of the myricetin glucuronide compound complexed with the enzyme and that of the hyaluronidase enzyme alone intersect each other at the beginning of the simulation (especially during 400 frames). This shows that the inhibition effect of myricetin glucuronide in this range is low, but then the halo form graph was above the apo form graph, and a noticeable difference occurred. This difference is an indication that the natural movement of the enzyme is disrupted by myricetin glucuronide. The halo form plot plateaued in the last half of the simulation (after 900 frames), keeping the RMSD value around 1.7 Å on average. The formation of a plateau level, i.e., a continuously repeating movement, is a good indicator for a long-lasting, effective and strong inhibition of enzyme activity. The fact that the halo form graph is much higher than the apo form graph indicates that the protein structure underwent a conformational change that interferes with its natural function.

When the RMSD graphs of the tyrosinase-myricetin glucuronide complex and the tyrosinase enzyme alone were examined, it was noticed that they differ from each other especially at the beginning of the simulation (up to frame 433). This indicates that myricetin glucuronide restricted the conformation of the protein. Over the simulation time, this difference disappeared, and the two graphs intersected each other. However, the halo form graph was slightly above the apo form, indicating enzyme inhibition. The simulation ended with a very stable plateau level. It was also observed that apo and halo forms graphs are sometimes intertwined. This is mainly due to the bulky protein structure of the tyrosinase enzyme. Bulky molecular structures increase the size effect, reducing the sensitivity of the graphs and increasing the margin of error (Sargsyan and Grauffel, 2017).

#### 3.4.2 Root-mean-square fluctuation (RMSF) analysis

**FIGURE 6:**
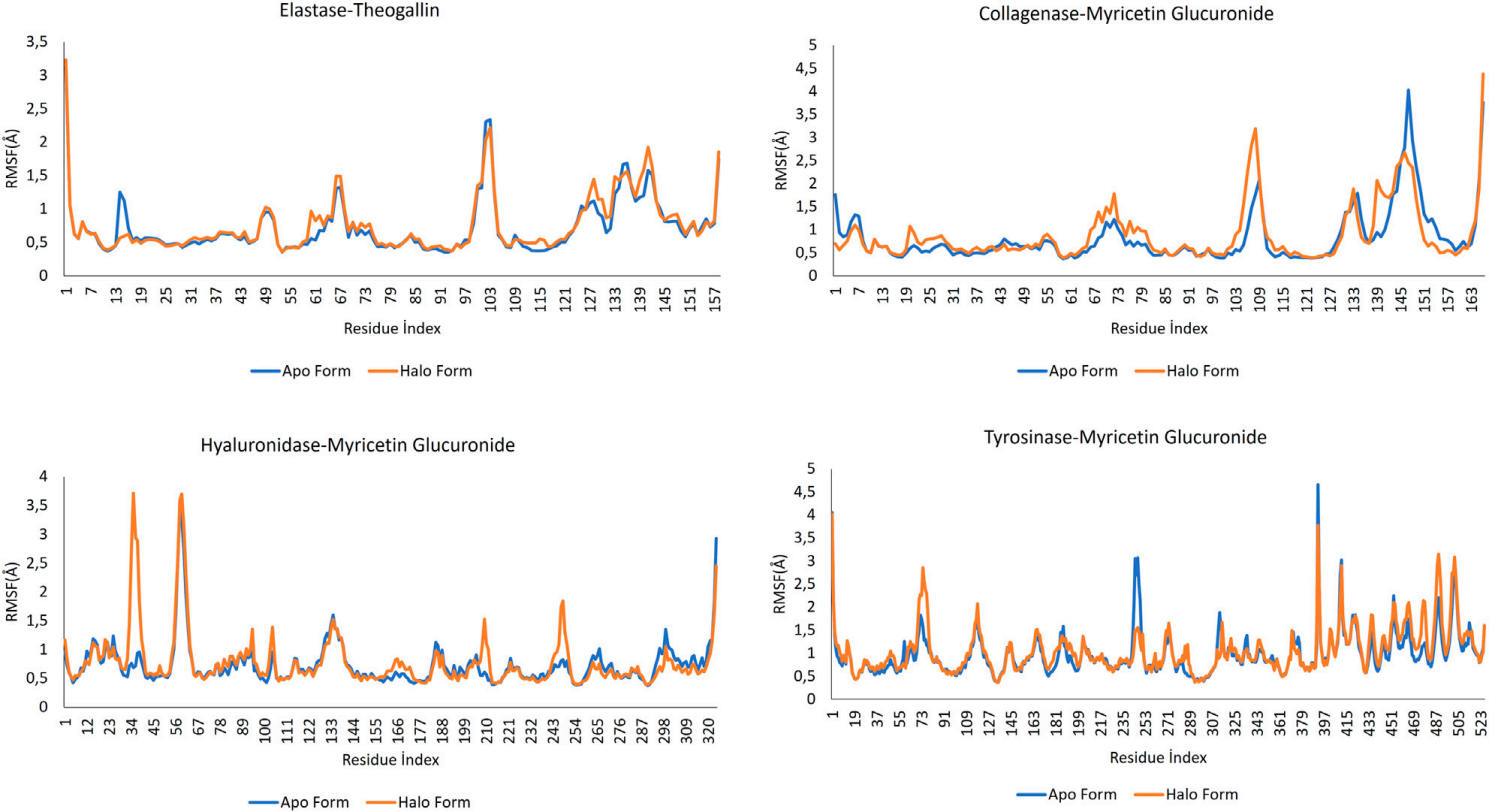
RMSF data for the complexes formed by the lead compounds and the different protein structures.

RMSF data measures the average deviation of the protein from the reference position. By analyzing this data, the fluctuating parts of the protein structure can be identified throughout the MD simulation. In all protein structures, the end regions showed high fluctuation, as expected. The parts forming the helix structures preserved their rigid structures. RMSF graphs of the apo and halo forms over the simulation time are shown in Figure 6.

Comparing the apo form of elastase and the halo form when elastase is complexed with theogallin, no significant differences in fluctuations were detected. Only between residue indices 13 and 17, the halo form appeared to dampen a fluctuation in the naturally functioning protein structure, which indicates no ligand contact in this region. However, other regions of the protein structure were highly fluctuating. Taking this together with the RMSD data, it could be concluded that the protein structure was strongly inhibited.

When the RMSF values of the collagenase-myricetin glucuronide complex and the enzyme’s apo form were analyzed, it was noticed that the halo form fluctuated more than the apo form in the region between 101 and 113-residue index. In other words, ligand contact increased the fluctuation in this region, and this could hinder the natural functioning of the enzyme. The opposite was observed with the residue of indices between 137 and 157. Myricetin glucuronide suppressed the fluctuation of the corresponding amino acids in the collagenase enzyme. These two contradictory data indicated that the functioning of the protein structure was completely disrupted, which could explain the strong inhibition of the plant extract against collagenase enzyme.

The apo form and halo form of hyaluronidase enzyme showed large differences in the local fluctuations. At the beginning, there were peaks in the region between 0-65 residue index where the halo form rose sharply. Contrary to the natural fluctuation of the protein structure, these peaks indicated disrupted function of the protein. These fluctuations were followed by other local fluctuations of the same type and nature.

Tyrosinase RMSF plot showed that this enzyme has many local fluctuations. Many sharply rising peaks were observed both in the apo form and in the halo form. Among these peaks, the apo form had a sharp peak within 235–261. The fluctuation of the halo form in this range was much less. This indicates that myricetin glucuronide could have the potential to dampen fluctuations and thus, a high potential to inhibit the enzyme activity.

#### 3.4.3 Generalized born surface area (MM-GBSA)

**FIGURE 7:**
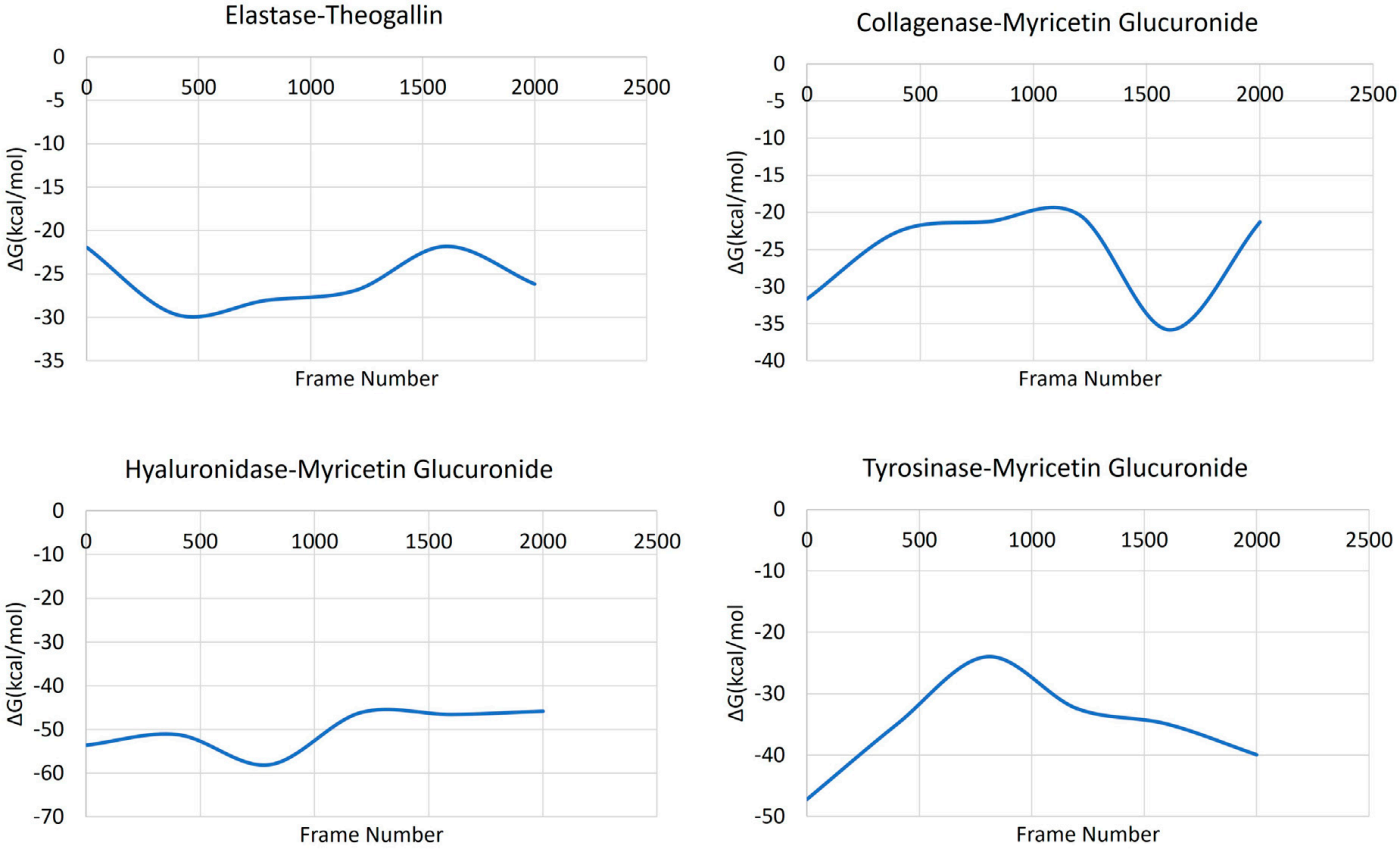
MM-GBSA plots of the ligand-protein complexes.

MM-GBSA calculations were successfully applied to estimate the binding free energy of the protein-ligand complexes, Figure 7. The obtained MM-GBSA data showed that all the complexes had low free energy that was maintained throughout the whole simulation time.

The MM-GBSA data of the complex formed by elastase and theogallin showed that the free energy remained constant in a certain range throughout the simulation. MM-GBSA data varied between −18 and −30 kcal/mol. Myricetin glucuronide, which was bound to collagenase enzyme with high affinity, started the MD simulation with a binding energy of −32 kcal/mol that increased later to −20 kcal/mol. In the last 1,000 frames (100 ns), where the MD simulation is stabilized, the binding energy decreased again to −36 kcal/mol and completed the molecular dynamics simulation at this range of values. The complex formed by hyaluronidase enzyme and myricetin glucuronide maintained its stable structure from the beginning until the end of the MD simulation. The hyaluronidase-myricetin glucuronide complex exhibited a free energy of around −45 kcal/mol throughout the simulation. Finally, the complex formed by the tyrosinase enzyme and myricetin glucuronide started the simulation with a free energy of −47 kcal/mol that rapidly increased and reached −24 kcal/mol within the first half of the simulation. Then after, the complex’s free energy decreased rapidly to reach −40 kcal/mol as the simulation formed a plateau level.

#### 3.4.4 Trajectory analysis

Trajectory analysis confirmed the strong interaction of the dominant compounds in *S. jambos* extract with the four aging promoting enzymes throughout the MD simulation. Theogallin clamped to the elastase structure and coordinated with the zinc atom via its carboxylate moiety. That was the most crucial factor to ensure that the compound remained attached to the protein structure throughout the simulation. The compound afforded conformational changes around this interaction throughout the MD simulation. The ester group and the six rings in theogallin played a role in the conformational changes of the compound. In particular, the six-ring bends and forms the basis of the conformational changes in the compound. Throughout the simulation, theogallin maintained its interactions with the protein structure and remained at the initial binding site.

Myricetin glucuronide is a compound with a rigid structure and maintained this property throughout the MD simulation of its complex with collagenase. The compound started the simulation by making important interactions within the enzyme. One of these important interactions is the coordination of the carboxylate moiety in the glucuronide group with the zinc ion. Among the residues involved in the coordination around the zinc ion was His222, which afforded pi-pi interaction with the C ring in myricetin glucuronide. This interaction continued throughout most of the simulation and occasionally the A ring was shown to participate in these interactions via pi-pi interaction. At 180 ns of the simulation, one of the hydroxyl groups in the A ring of the myricetin glucuronide afforded a hydrogen bond with the Glu223 residue. This interaction is important in terms of being in the last 100 ns when the simulation stabilized, which reveals a high potential for the interaction to continue.

Similarly, myricetin glucuronide started the MD simulation by forming coordination between the carbonyl oxygen in the glucuronide group and the copper ion in the tyrosinase enzyme, an interaction that continued throughout the simulation and was the main interaction that linked the compound to the protein structure. The hydroxyl groups on the B ring of the compound stabilized this part of the molecule by making multiple hydrogen bonds with Glu322 from the beginning to the end of the simulation. With these interactions, the simulation ended with no conformational changes except for few fluctuations in the conformation of the myricetin glucuronide, where it was initially bound.

With respect to hyaluronidase enzyme, myricetin glucuronide started by the reactive groups in the B ring and the glucuronide group forming hydrogen bonds with the protein structure. The compound had multiple interactions (many hydrogen bonds and salt bridges) with residues Glu113, Arg274 and Ser303. It remained inside its initial binding site without significant conformational changes throughout the simulation and maintained the same interactions.

### 3.5 *In vitro* antioxidant and anti-aging activities

Polyphenols have been used for centuries in preventive medicine and constitute one of the most representative families of secondary metabolites in the plant kingdom (Wyk et al., 2015). They are well-documented for their strong antioxidant and cytoprotective properties with the potential to be used as value-added ingredients in the development of cosmetic products. Indeed, herbal cosmeceuticals are taking part of the cosmetic market, and various plant-derived compounds are being successfully commercialized (Petruk et al., 2018). The extract exhibited significant antioxidant potential in the DPPH assay compared to quercetin, used as a reference antioxidant (Table 4). This strong antioxidant effect could be related to the richness of the plant in flavonoids and high phenolic contents. Similar activities were observed using the methanol leaf extracts from Indonesia, ethanol plant extracts from South Africa and ethanol leaf extract from India (Ramadhania et al., 2017; Twilley et al., 2017; Rajkumari et al., 2018a). Potent antioxidant activity was also observed in the FRAP assay (Table 4). It is well established through the *in vitro* assays that *S. jambos* has substantial antioxidant potential (Ticona et al., 2021). This was also supported by the studies of Sobeh et al., who reported that the Egyptian *S. jambos* harbors plenty of phenolic compounds such as flavonol glycosides, flavones ellagitannins, and phenolic acids, which are known to be endowed with strong antioxidant properties (Sobeh et al., 2018a).

**TABLE 4:** Total phenol content, antioxidant, and anti-aging activities of *S. jambos* leaf extract.

Parameter	SJWE	Quercetin	Kojic acid
Polyphenols (mg GA/g extract)	230.17 ± 1.12	–	–
Flavonoids (mg QE/g extract)	18.96 ± 0.51	–	–
	**IC**_**50**_ **(µg/mL)**		
DPPH	10.1 ± 0.24	1.07 ± 0.01	–
FRAP	20.34 ± 0.41	24.04 ± 1.23	–
Elastase	18.74 ± 3.2	–	21.60 ± 0.9
Tyrosinase	3.49 ± 0.2	–	14.46 ± 0.6
Hyaluronidase	20.61 ± 1.2	–	9.00 ± 0.9
Collagenase	21.57 ± 2.5	24.83 ± 1.8	–

SJWE: *S. jambos* water extract; g GA/g of extract: g of gallic acid/g of extract; g QE/g of extract g of quercetin/g of extract.

IC_50_: Half maximal inhibitory concentration.

The cosmeceutical properties of *S. jambos* aqueous extract were assessed, *in vitro*, by evaluating its potential to inhibit skin-aging-related enzymes, namely, collagenase, elastase, tyrosinase and hyaluronidase. As discussed earlier, both collagen and elastin are essential to maintain the flexibility, plumpness, integrity, and elasticity of the skin. Collagen secures the tensile strength of the skin, whereas elastin provides the elastic recoil characteristics (Horng et al., 2017). Hyaluronic acid, on the other hand, promotes skin rejuvenation and reduces the permeability of the extracellular fluid (Jegasothy et al., 2014). In the same context, overproduction of melanin is associated with skin disorders such as melasma, freckles, and hyperpigmentation leading to premature skin aging (Chatatikun et al., 2019). Consequently, inhibition of these enzymes’ activity is an effective strategy to manage skin aging for aesthetic purposes.

The extract exhibited strong inhibitory activity against tyrosinase and elastase enzymes (IC_50_ = 3.49 and 18.74 μg/mL, respectively) and lower inhibitory activity against hyaluronidase and collagenase enzymes (IC_50_ = 20.61 and 21.57 μg/mL, respectively), when compared to the reference inhibitors kojic acid and quercetin (Table 4). Noteworthy, our *in vitro* results came, largely, in alignment with the *in silico* findings, where molecular docking showed that all the docked extract’s compounds had better binding affinity than kojic acid towards tyrosinase and elastase enzymes, while not all of them surpassed the binding affinity of the reference inhibitor upon docking to hyaluronidase and collagenase enzymes. Previous studies reported that *Syzygium* species possess cosmeceutical properties as they are rich in various flavonoids, which are strong antioxidants endowed with anti-aging properties and skin protective activity against UV radiations (Hyun et al., 2020).

### 3.6 Biocompatibility of *S. jambos* leaves extract

**FIGURE 8:**
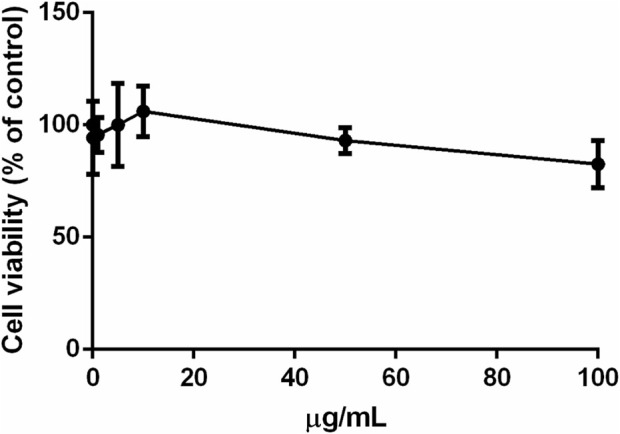
Effect of *S. jambos* leaf extract on HaCaT cells viability after 48 h incubation.

The biocompatibility of the leaf aqueous extract of *S. jambos* was studied on immortalized human keratinocytes (HaCaT). Cells were treated with increasing concentrations of the extract (0.1–100 μg/mL) for 48 h and cell viability was assessed by MTT assay. As shown in Figure 8, no cytotoxic effect was observed at any of the concentrations tested up to 48 h incubation, thus confirming the biocompatibility of the tested extract to the human keratinocytes.

### 3.7 Cell-based antioxidant activity

**FIGURE 9:**
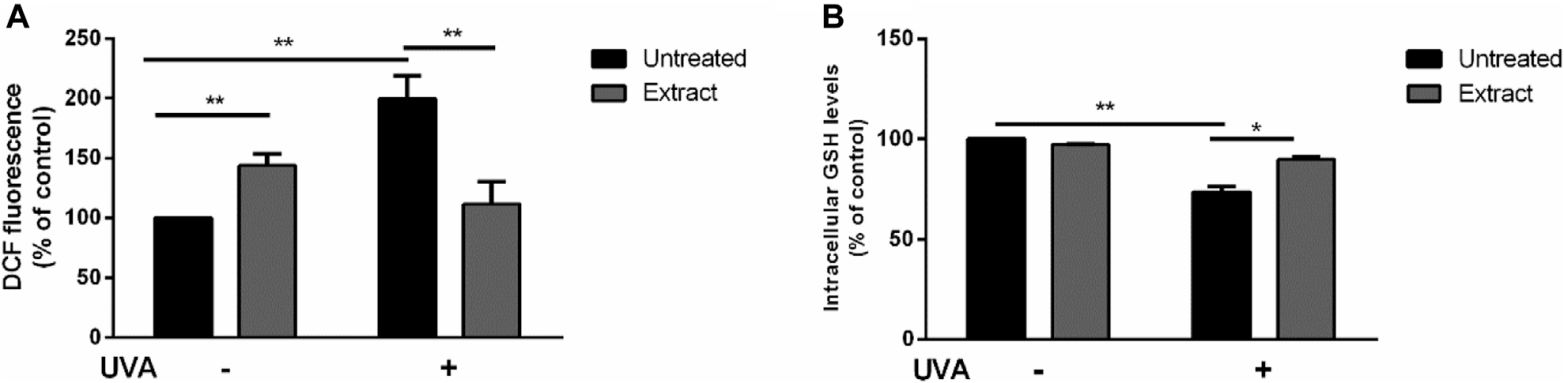
Protective effect of *S. jambos* leaf extract against UVA injury on HaCaT cells. Cells were preincubated in the presence of 50 μg/mL of *S. jambos* extract, prior the UVA irradiation (100 J/cm^2^). Black bars are referred to untreated cells and grey bars to cells incubated with the extract for 2 h, in the presence (+) or absence (−) of UVA irradiation, **(A)** Determination of intracellular ROS levels by DCFDA assay; **(B)** Determination of GSH levels by DTNB assay. All treated groups were analyzed by one-way analysis of variance followed by Bonferroni (*post hoc*). * indicates *p* < 0.05, ** indicates *p* < 0.01.

The antioxidant activity of *S. jambos* aqueous extract was also studied on a well-established cell-based system. HaCaT cells were cultured with 50 μg/mL of the extract for 2 h, and then subjected to UVA-induced oxidative stress, as described in Materials and Methods section. As shown in Figure 9A, an increase in ROS production was observed when cells were incubated with *S. jambos* aqueous extract in the absence of any UVA stress, which may suggest a prooxidant effect of the extract at the tested concentration. However, the extract was able to inhibit the deleterious effects of UVA, as no increase in ROS content was observed when cells were preincubated with the extract before inducing oxidative stress. Similar findings were obtained from *S. samarangense* leaves extract and its individual components (myricitrin and 3,5-di-*O*-methyl gossypetin) (Sobeh et al., 2018b; Sobeh et al., 2019). To further confirm the protective effect, intracellular GSH levels were measured. As shown in Figure 9B, in the absence of any treatment (black bars), a significant (*p* < 0.01) decrease in GSH levels was observed when cells were irradiated with UVA; whereas *S. jambos* extract was able to inhibit GSH oxidation (grey bars) upon UVA exposure. Likewise, the aqueous extract of *S. jambos* resulted in a significant antioxidant potential, which could be due to the free radical scavenging capacity of the phytocompounds that the plant contains.

### 3.8 Antibacterial activities

**FIGURE 10:**
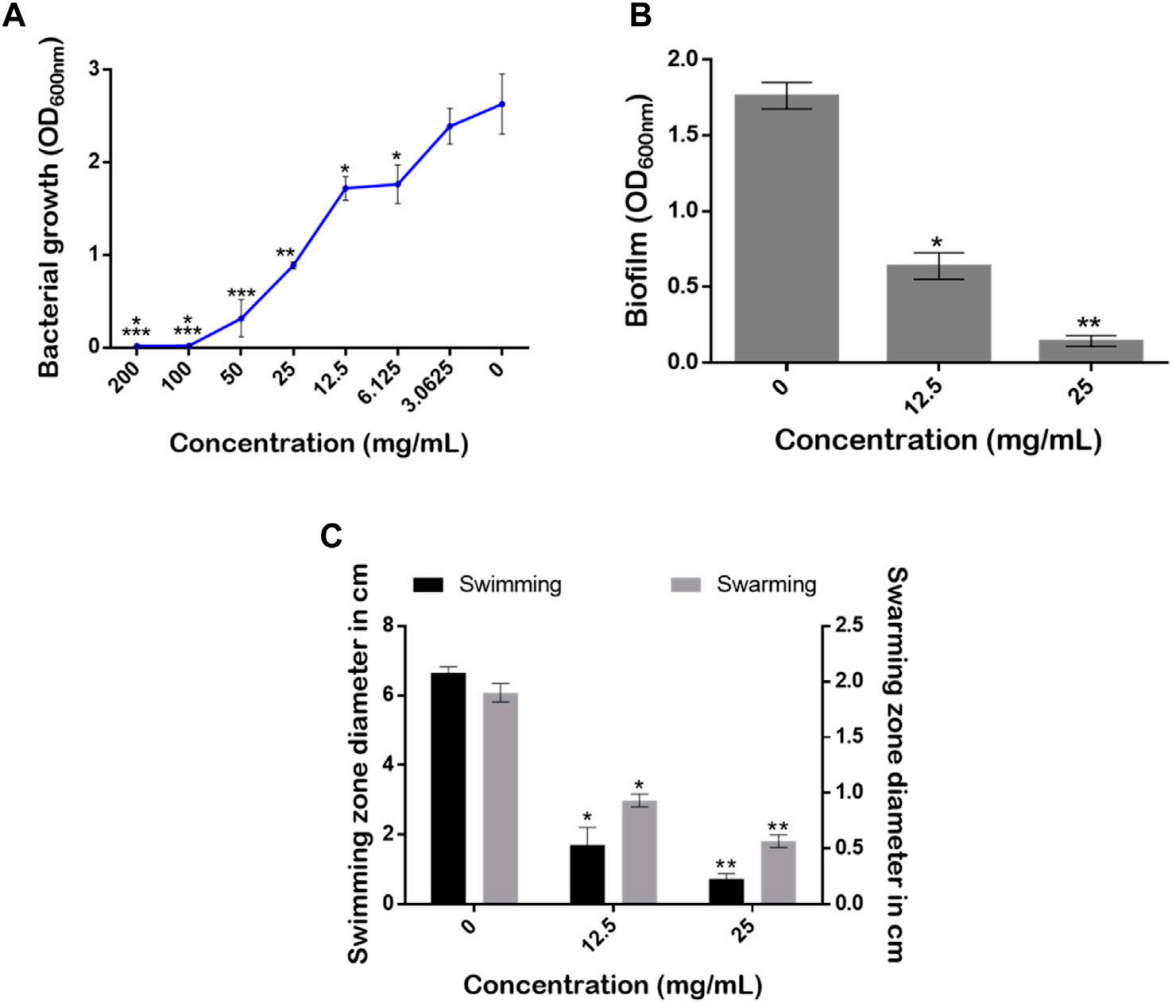
Effect of *S. jambos* water extract concentrations on **(A)** bacterial growth rate, **(B)** biofilm production, and **(C)** the swimming and swarming mobilities of *Pseudomonas aeruginosa* grown in the absence and presence of the extract tested at 12.5 mg/mL (1/8 MIC) and 25 mg/mL (1/4 MIC). Asterisks (*) indicate a significant difference at *p* < 0.05 using Tukey test.

*Pseudomonas aeruginosa* is a major clinical opportunistic bacterium responsible for up to 57% of total nosocomial infections (Krishnan et al., 2012). Many of the infections caused by *P. aeruginosa* are characterized by cutaneous manifestations (Wu et al., 2011). Thus, the effects of the aqueous extract from *S. jambos* leaves on the growth rate, biofilm, and swarming and swimming mobilities of *P. aeruginosa* were evaluated, Figure 10. According to the established criteria, above 50% inhibition is regarded as a good anti-biofilm activity. The results revealed that treatment of the culture media with the leaf extract induced a complete inhibition of the bacterial growth at MIC = 100 mg/mL (Figure 10A).

Although conventional strategies aimed at reducing microbial infections are efficacious, targeting virulence factors rather than their survival and growth appears to be an attractive approach. This approach may lead to reduced pathogenicity of pathogens and a reduction in the development of resistance (Silva et al., 2016). Indeed, the ability of a pathogenic bacterium to form biofilm communities and to move across surfaces is very important for virulence determination (Kearns, 2010; Rajkumari et al., 2018b). Interestingly, our extract at sub-inhibitory concentrations (1/8 MIC = 12.5 mg/mL and 1/4 MIC = 25 mg/mL) significantly reduced the amount of the biofilm produced with inhibition percentages of 41.17% and 88.23%, respectively (Figure 10B). On the other hand, it is well established that bacterial mobility over biological surfaces enables pathogenic strains to move, adhere and migrate from infection sites (Kearns, 2010). Here, the extract counteracted the swimming and swarming mobilities of the pathogenic strain in a dose-dependent manner using the two sub-MICs. The mobilities of *P. aeruginosa* monitored on plates revealed that, compared to media without the extract (0 mg/mL), *S. jambos* aqueous extract significantly reduced the swimming mobility by 74.43% and 89.39% using 1/8 (12.5 mg/mL) and 1/4 (25 mg/mL) MICs, respectively. As for the swarming motility, the extract at 25 mg/mL reduced the swarming diameter by up to 70.52% while the other 12.5 mg/mL concentration resulted in 52.63% inhibition compared to the control (Figure 10C). In conclusion, the extract inhibited bacterial growth, reduced the biofilm formation, and counteracted *P. aeruginosa* mobility in a dose-dependent manner.

Several extracts from *S. jambos* showed pronounced antimicrobial activities against many pathogenic strains such as *Staphylococcus aureus*, *Escherichia coli*, and *Klebsiella pneumonia* (MIC = 0.5–2.5 mg/mL) (Ochieng et al., 2022). A study showed that the leaf and bark extracts of *S. jambos* demonstrated up to 70% of antibiotic-regulating activity against *P. aeruginosa* (Wamba et al., 2018). This antimicrobial activity could be attributed to the effect of phenolic compounds that the plant contains, such as rutin, quercetin, and 3,4,5-trihydroxybenzoic acid, among others (Ghareeb et al., 2017). Moreover, Famuyide et al. (2019) showed that *Syzygium* species, such as *S. legatii* and *S. masukuense* were the most performant in inhibiting both Gram-negative and Gram-positive bacteria with MIC values ranging from 0.04 to 0.08 mg/mL (Famuyide et al., 2019). The plant extracts were also potent in reducing bacterial biofilm formation and in destroying the formed bacterial biofilms (Morán et al., 2014). However, future antimicrobial assays should investigate the extract’s efficacy in animal models as well as against other infectious strains. It is also imperative to assess safety profiles and potential side effects *in vivo*. Moreover, successful translation into clinical applications requires careful consideration of dosage, delivery methods, and interactions with potential existing treatments. Based on these findings, *S. jambos* may be a promising dermacosmeceutical and antimicrobial agent of powerful antioxidant potential for retarding skin aging and combating skin infections.

## 4 Conclusion

The present study examined the dermatological and skin anti-aging activities of an aqueous extract from *S. jambos* leaves. The LC-MS/MS data revealed that the extract contained different phenolic metabolites, such as gallic acid, quercetin, myricetin and kaempferol derivatives. Moreover, the plant leaves contained moderate levels of nitrogen, potassium, magnesium, manganese, zinc, relatively low contents of phosphorus and copper and high calcium and iron concentrations. The obtained results support the conventional potential use of *S. jambos* leaves as a source of high value-added agents having skincare benefits. These include antioxidant, anti-aging, antibacterial and anti-biofilm activities while being biocompatible with human keratinocytes. Nevertheless, further studies are highly needed to uncover the components of *S. jambos* extract that are responsible for the observed beneficial dermatological effects, including their corresponding cellular and molecular mode of action as well as detailed toxicity studies on bacterial cells.

## Data Availability

The original contributions presented in the study are included in the article/Supplementary material, further inquiries can be directed to the corresponding author.

## Abbreviations

CV, Crystal Violet; DPPH, 2,2-DiPhenyl-1-PicrylHydrazyl; DW, Dry Weight; ECM, Extracellular Matrix; FRAP, Ferric Reducing Antioxidant Power; GBSA, Generalized Born Surface Area; GSH, Glutathione; H2-DCFDA, 2′,7′-dichlorodihydrofluorescein diacetate; HaCaT, Human keratinocytes cells; HPLC-PDA-MS/MS, High-Performance Liquid Chromatography-PhotoDiode Array detector coupled to Mass Spectrometry; ICP-EOS, Inductively Coupled Plasma Optical Emission Spectroscopy; MD, Molecular Dynamics; MIC, Minimum Inhibitory Concentration; MTT, 3-(4,5-dimethylthiazol-2-yl)-2,5-diphenyltetrazolium bromide); RMSD, Root-Mean-Square Deviation; RNS, Reactive Nitrogen Species; ROS, Reactive Oxygen Species; UVA, UltraViolet A.
